# THE STRENGTH OF WEAKER TIES: HAVE WE BEEN IGNORING A RESOURCE FOR AGING ADULTS?

**DOI:** 10.1093/geroni/igz038.2918

**Published:** 2019-11-08

**Authors:** Katherine L Fiori, Oliver Huxhold, Noah J Webster, Toni C Antonucci

**Affiliations:** 1 Adelphi University, Garden City, New York, United States; 2 German Centre of Gerontology, Berlin, Berlin, Germany; 3 Institute for Social Research Room, University of Michigan, Ann Arbor, Michigan, United States

## Abstract

The purpose of this study was to examine links between changes in social ties (close ties and weaker ties) and changes in positive and depressed affect across three waves in a large, representative sample of U.S. adults aged 40 and over (N = 802). Using trivariate dual-change score models, we found that a greater number of weaker ties was associated with higher numbers of close ties over time, and that the number of weaker ties was more strongly predictive of positive age-related changes in both aspects of well-being (i.e., less depressed affect and more positive affect) than the number of close ties. Our findings imply that focusing investment on the outer circles may have the unintended benefit of compensating for losses in the inner circle, and that contrary to popular theoretical orientations, weaker ties may offer older adults an avenue for both promoting positive affect and decreasing negative affect.

